# A Bibliometric Analysis of Publications on Endoscopic Ultrasound

**DOI:** 10.3389/fmed.2022.869004

**Published:** 2022-03-29

**Authors:** Xiaoli Chen, Huiqin He, Xin Chen, Xueqin Chen, Zhenzhen Wen, Mengque Xu, Yanfei Fang, Xingkang He

**Affiliations:** Department of Gastroenterology, Sir Run Run Shaw Hospital, Zhejiang University Medical School, Hangzhou, China

**Keywords:** endoscopic ultrasound, bibliometric analysis, bibliometrix, VOSviewer, gastroentergology

## Abstract

**Background:**

Over the past 40 years, endoscopic ultrasound (EUS) has become a safe and effective tool for both diagnostic and therapeutic applications. A growing number of articles have been published annually. We aimed to explore global scientific outputs and hotspots of EUS published by different countries, organizations, and authors.

**Methods:**

The global literature regarding EUS during the 1900–2020 period was identified from the Web of Science (WOS) Core database. “Bibliometrix” and software VOSviewer were applied to perform bibliometric analysis.

**Results:**

The annual growth rate of publications from 1980 to 2020 was around 16% and the number of EUS-related articles had experienced a sudden increase in the last decade. Bhutani MS was the most productive author over the past years, with 94 publications. Hawes RH had the highest number of citations, with 6,034 citations. The United States and institutions from United States dominated the EUS research. Among the journals, GASTROINTESTINAL ENDOSCOPY published the highest number of articles, followed by ENDOSCOPY. The majority of top 10 frequently cited references were cited more than 200 times. Carcinoma, diagnosis, fine-needle-aspiration, cytology, and pancreatitis were the important keywords in co-occurrence analysis of keywords. Recent studies focused more on tissue acquisition, size of the needle, lumen-apposing metal stent, and fine-needle- biopsy.

**Conclusion:**

Research on EUS has significantly increased in the last decade globally and it will continue to increase. Active collaboration among different authors and countries was observed in the EUS field. Tissue acquisition, size of the needle, apposing metal stent, and fine-needle-biopsy might be the latest research frontiers and should receive more attention.

## Introduction

Endoscopic ultrasound (EUS), a combination of endoscopy and ultrasound, was initially developed in the 1980s for differential diagnosis of various gastrointestinal benign and malignant conditions (1). EUS allowed the gastroenterologists to clearly visualize structures of the gastrointestinal tract (2). With the advent of the fine needle aspiration (FNA) in 1991, EUS became a powerful and potential tool in diagnostic and therapeutic management (3). Over the last 40 years, EUS has been expanded and developed for managing hepatobiliary, pancreatic, lung, and mediastinal diseases due to progression of innovation of echoendoscopic equipment (4, 5). It is widely used to diagnose or stage malignancies and acquire tissues. Broadly, EUS can be classified into radial and linear EUS. Radial EUS is primarily used for the diagnostic purpose, and linear EUS also facilitates image-guided tissue sampling and intervention. Due to elimination of the effect of intestinal gas and fat, EUS provides an added advantage, such as high resolution imaging for the identification and evaluation of small pancreatic masses and cysts. Most importantly, EUS has become indispensable in the staging of a variety of upper gastrointestinal tract tumors. Nowadays, EUS has increasingly shifted from diagnostic roles to therapeutic management, such as EUS-guided sampling, EUS-guided drainage of biliary, gallbladder, pancreatic duct, non-pancreatic abdominopelvic collections, and EUS-guided ablation. The future of EUS-guided interventions seems to be bright and promising. Due to its minimally invasive technique and good accuracy, EUS has increasingly been used in the field of gastroenterology and has attracted growing interest (5–7). EUS plays important roles in the diagnosis of pancreatitis, submucosal lesions, and pancreatic lesion, staging of gastrointestinal cancer, biopsy, drainage of pseudocysts, and celiac plexus neurolysis. Despite an increasing research output in the EUS field, little is known about the scientific production related to EUS and a bibliometric analysis has not been published to date.

Bibliometric analysis is a statistical method to analyze the scientific publications related to a special topic. It is an important tool to quickly acquire scientific information and assess the key areas of research and new potential trends in future studies. Therefore, a bibliometric analysis is urgently needed to provide a broad understanding of EUS and future research direction. In the present study, we analyzed the EUS-related articles retrieved from the Web of Science (WOS) Core database to evaluate the existing literature with respect to the authors, year, journals, countries, institutions, keywords, and references. We aimed to provide a broad understanding of EUS-related key topics and future directions.

## Materials and Methods

A comprehensive literature search was performed in the WOS Core database (Clarivate Analytics, United States), which is considered as the most appropriate database for bibliometric analysis (8). The search terms were endoscopic ultrasound, EUS, and their synonyms, including “endoscopic ultrasonography.” The detailed search strategies used were as follows: TS = (“endoscopic ultrasound”) OR TS = (“endoscopic ultrasonography”) OR TS = (“ultrasonic fiberendoscope”) OR TS = (“ultrasonic tomography”). The period of literature search was from 1900 to 2020 and document types were restricted to original articles. We performed literature search and downloaded raw data on a single day, April 1, 2021, to reduce bias due to frequent database renewal. There was no limitation on the language during the initial process of retrieval. Finally, non-English literature was excluded from our final analysis. The detailed flow chart of enrollment is summarized in Supplementary Figure 1.

Raw data of included references were initially downloaded from WOS Core database as the plain text file. We extracted following information, such as the title, author, institution, country, publication year, references, and keywords, from raw data and saved it in a TXT format. The data was further imported into “Bibliometrix” (R package) (9) in Rstudio and analyzed annual publications, trends, distribution and frequency of the author, journal, country, and institute. The data obtained from the WOS were converted into VOSviewer version 1.6.16 (Leiden University) (10) for analysis of co-authorship, co-cited references, and co-occurrence of keywords. The standard weight attribute was applied to define “links attribute” and “total link strength attribute.” In particular, we also applied co-occurrence of keywords in the order of year to detect trends and hotspots. In the network, different nodes in the maps represented elements such as author, institution, journal, country, keywords and references. The size of nodes represented weight or frequence of author, institution, journal, country, keywords and references. The links between different nodes represented relationships among co-occurrence or co-citations of author, institution, journal, country, keywords and references. The default threshold was applied in the VOSviewer software.

## Results

We performed a comprehensive literature search and identified 9,335 articles published over the past 40 years. Of the 9,335 papers, 8,796 (94.22%) were published in English, 218 (2.34%) in German, 195 (2.09%) in French, 88 (0.94%) in Spanish, and 7 (0.08%) in Hungarian language. After exclusion of duplicated articles, we finally included 8,791 articles published in English for our analysis.

### Publication Trend

**FIGURE 1:**
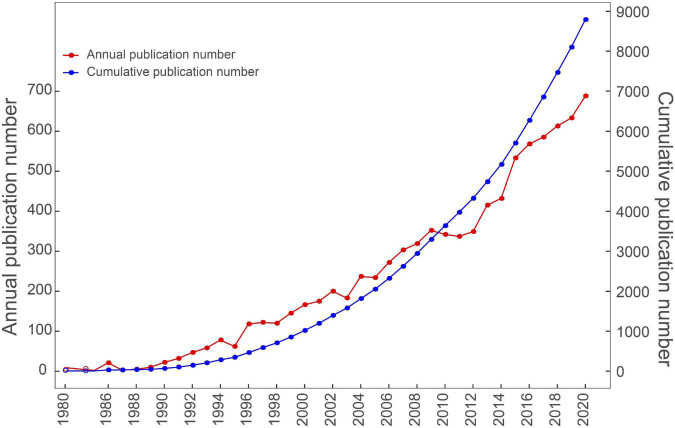
The pattern of the annual and cumulative number of publications in the period from 1980 to 2020.

The first article in the field of EUS was published in 1980 (11). Strohm et al. first used an ultrasonic fiberendoscope to examine patients with biliary, pancreatic, and hepatic disorders (11). The number of publications gradually and generally increased in the subsequent years (Figure 1). Before 2002, the annual number of publications was less than 200 (Figure 1). From 1995 to 2012, the annual number of articles gradually increased from 62 in 1995 to 349 in 2012. After 2012, the annual number of articles increased rapidly, from 415 in 2013 to 688 in 2020. The annual growth rate of publications from 1980 to 2020 was around 16%. From 2011 to 2020, there were a total of 5,490 EUS-related articles, accounting for 62.45% of overall publications over the past 40 years.

### Analysis of the Author and Co-authorship

**FIGURE 2:**
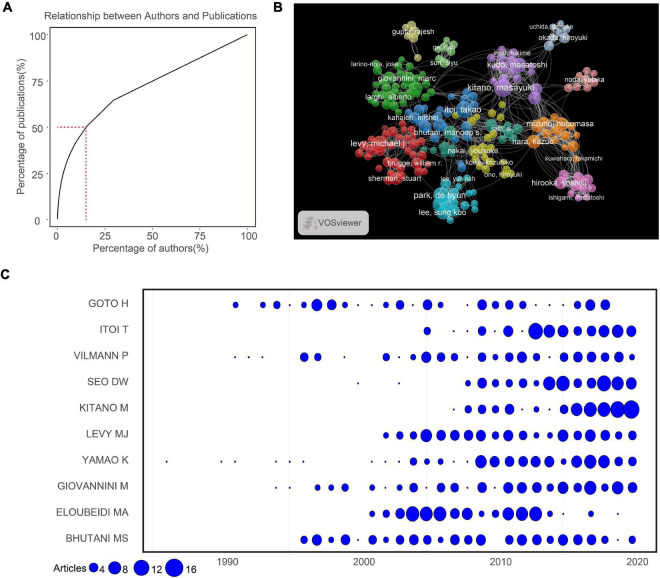
**(A)** Relationship between the total number of authors and publications. **(B)** The network map of authors for EUS research. **(C)** The number of publications for the top 10 productive authors per year.

A total of 28,420 authors were obtained in the 8,795 articles. As shown in Table 1, the top 10 authors contributed 829 (9.43%) articles. Manoop S Bhutani (94, 1.07%) produced the highest number of articles, followed by Eloubeidi MA (91, 1.04%), Giovannini M (89, 1.01%), Yamao K (88, 1.00%), and Levy MJ (84, 0.96%). Less than 20% of authors contributed around 50% of all publications (Figure 2A). Our co-authorship analysis of authors showed that 275 of 28,420 authors had published at least 15 papers, and the largest set of associated authors consisted of 35 authors in the red cluster (Figure 2B). We also calculated the top 10 authors’ productions over time and found that Itol T, Seo DW, Kitano M, Levy MJ, Yamao K, and Giovannini M produced more articles recently (Figure 2C). Vilmann P, Goto H, and Bhutani MS produced more articles during the early history of EUS, suggesting that these authors might be pioneers in this field. Among the most frequently cited authors, Hawes RH ranked first in the list, with 6,034 citations, followed by Wiersema MJ (5,480 citations), Giovannini M (5,456 citations), Vilmann P (4,719 citations), and Palazzo L (4,419 citations) (Table 1).

**TABLE 1:** The top 10 productive authors and the most-cited authors in the field of EUS.

Author	Articles (n)	Author	Total citation (n)
BHUTANI MS	94	HAWES RH	6,034
ELOUBEIDI MA	91	WIERSEMA MJ	5,480
GIOVANNINI M	89	GIOVANNINI M	5,456
YAMAO K	88	VILMANN P	4,719
LEVY MJ	84	PALAZZO L	4,419
KITANO M	81	BRUGGE WR	4,374
SEO DW	78	ELOUBEIDI MA	4,287
VILMANN P	76	SHERMAN S	4,060
ITOI T	75	HOFFMAN BJ	3,816
GOTO H	73	SIVAK MV	3,256

### Analysis of the Institution and Country

**FIGURE 3:**
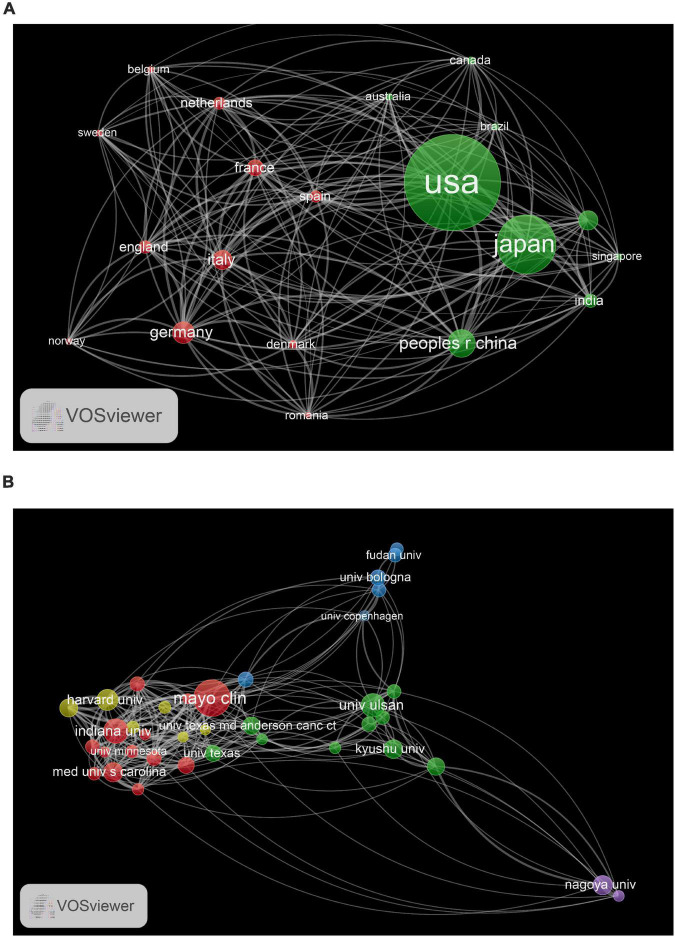
**(A)** The network map of countries for EUS research. **(B)** The network map of institutions for EUS research.

A total of 4,876 institutions from eighty countries contributed to the publications on EUS research. The top 10 countries contributed 4,797 (80.80%) articles, and the top five countries were United States (2,576), Japan (1,578), China (751), Korea (420), and Germany (405) (Table 2). A network map was created for the co-authorship analysis of countries, and United States, Japan, and China were the top three large nodes, representing the most productive countries in this field (Figure 3A). The top 10 institutions contributed around 24% of EUS-related publications and most of them were from United States, except for UNIV ULSAN (South Korea), AICHI CANC CTR HOSP (Japan), KYUSHU UNIV (Japan), UNIV AMSTERDAM (Netherlands), and SEOUL NATL UNIV (South Korea) (Table 2). Among the top 10 institutions, MAYO CLIN (265) published the highest number of articles, followed by UNIV TEXAS MD ANDERSON CANC CTR (223), UNIV ULSAN (204), and INDIANA UNIV (204). The co-authorship analysis of institutions was performed and a network map was created by VOSviewer. As shown in Figure 3B, there were four clusters with different colors among the top 100 institutions. This suggested that active collaborations were common among the top institutions (Figure 3B), especially for institutions in the same cluster.

**TABLE 2:** The top 10 countries and institutions contributed to publications of EUS research.

Country	Articles (n)	Author	Articles (n)
USA	2,576	MAYO CLIN	265
JAPAN	1,578	UNIV TEXAS MD ANDERSON CANC CTR	223
CHINA	751	UNIV ULSAN	204
KOREA	420	INDIANA UNIV	204
GERMANY	405	AICHI CANC CTR HOSP	183
ITALY	366	KYUSHU UNIV	183
FRANCE	286	UNIV TEXAS	176
UNITED KINGDOM	276	UNIV AMSTERDAM	167
INDIA	242	HARVARD UNIV	155
NETHERLANDS	212	SEOUL NATL UNIV	155

### Analysis of Journals

**FIGURE 4:**
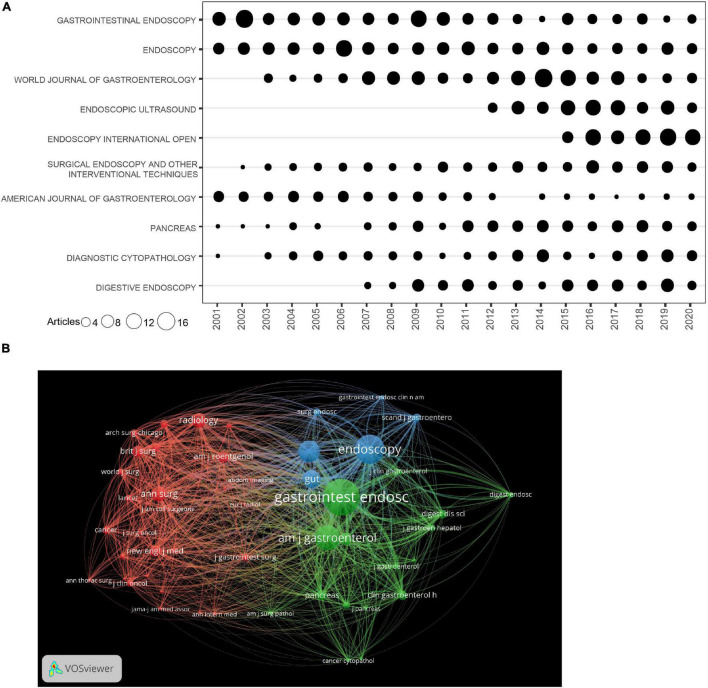
**(A)** The number of publications for the top 10 journals per year. **(B)** The network map of journals for EUS research.

There was a total of 865 journals, and Table 3 presents the top 10 journals in the EUS field. Top 10 journals contributed a total of 1,704 (25.76%) papers. GASTROINTESTINAL ENDOSCOPY ranked first, followed by ENDOSCOPY, WORLD JOURNAL OF GASTROENTEROLOGY, ENDOSCOPIC ULTRASOUND and ENDOSCOPY INTERNATIONAL OPEN (Table 3). Among them, six were from United States, two from Germany, one from Japan, and one from India (Table 3). The impact factor of three journals was greater than 6 and most of the journals were ranked in the Q1 or Q2 category, indicating high quality of the articles (Table 3). Figure 4A displays the trend of annual publications (top 10 journals) during 2001–2020. GASTROINTESTINAL ENDOSCOPY, ENDOSCOPY, and WORLD JOURNAL OF GASTROENTEROLOGY produced considerable and consistent publications in this field. DIGESTIVE ENDOSCOPY, ENDOSCOPIC ULTRASOUND, and ENDOSCOPY INTERNATIONAL OPEN were relatively new journals that developed very quickly. The co-authorship analysis of journals was performed, and a network map was constructed. As shown in Figure 4B, institutions in the same cluster were closely involved in active collaborations.

**TABLE 3:** The top 10 journal contributed to publications of EUS research.

Journal	Articles (n)	Impact factor(2019)	Quartile in category
GASTROINTESTINAL ENDOSCOPY	527	6.89	Q1
ENDOSCOPY	481	7.34	Q1
WORLD JOURNAL OF GASTROENTEROLOGY	305	3.66	Q2
ENDOSCOPIC ULTRASOUND	185	4.49	Q2
ENDOSCOPY INTERNATIONAL OPEN	177	/	/
SURGICAL ENDOSCOPY AND OTHER INTERVENTIONAL TECHNIQUES	173	3.15	Q1
AMERICAN JOURNAL OF GASTROENTEROLOGY	169	10.17	Q1
PANCREAS	165	2.92	Q3
DIAGNOSTIC CYTOPATHOLOGY	155	1.23	Q3
DIGESTIVE ENDOSCOPY	155	4.774	Q1

### Co-cited Reference and Keyword Co-occurrence Cluster Analysis

**FIGURE 5:**
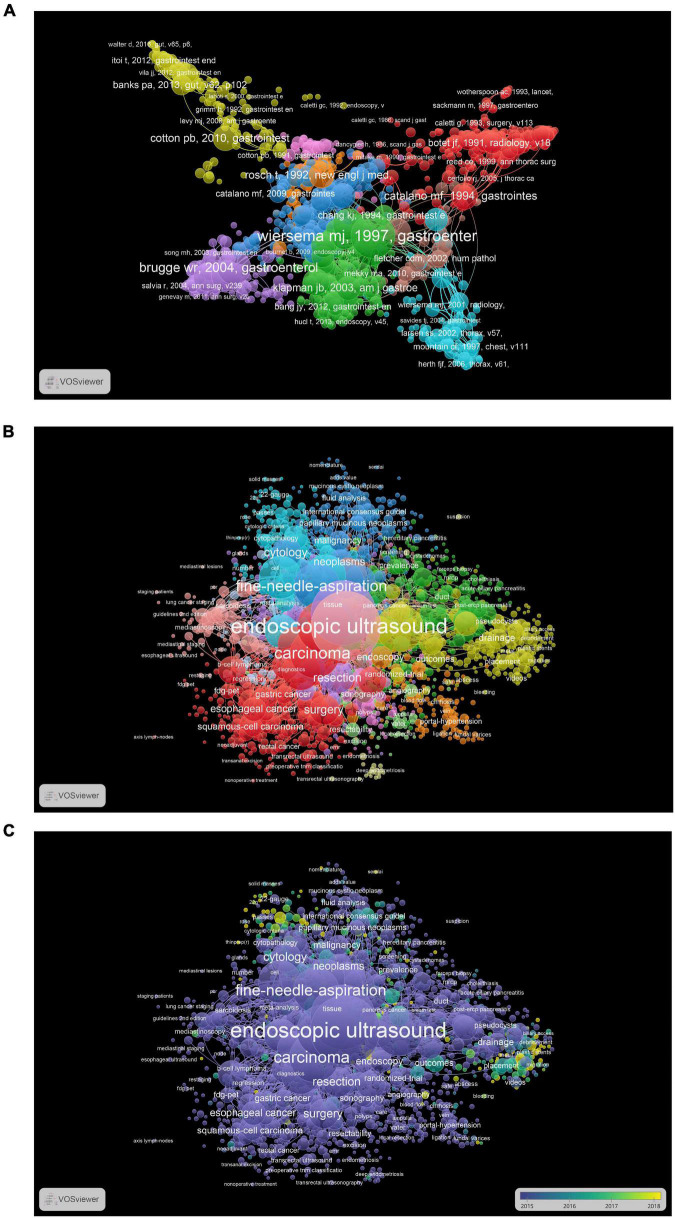
**(A)** The network map of publications for EUS research. **(B)** The network map of keywords for EUS research. **(C)** The network map of keywords for EUS research during 2015–2018, different colors indicating different years.

Table 4 presents the top 10 most frequently cited articles. As shown in Table 4, one reference was cited more than 400 times (12), and eight references were cited between 200 and 300 times (13–20). Through the co-citation analysis of references, we explored the most important and influential references for EUS studies. Cluster analysis of co-cited references was performed, and the network map was created for visualization. Figure 5A depicts nine clusters and the red cluster had the highest number of references, followed by green and blue clusters. A total of 13,711 keywords were extracted from publications and keywords with co-occurrences more than 100 were generated for the net map (Figure 5B). Cluster red included keywords related to different types of cancer (gastric cancer, esophageal cancer, rectal cancer, and lymphoma). Cluster yellow contained therapy-related keywords, including drainage, debridement, biliary access, and plastic stents. Carcinoma, diagnosis, fine-needle-aspiration, cytology, and pancreatitis were the important keywords in the co-occurrence analysis of keywords. Figure 5C shows the network map of the trend topics according to the keywords used between 2015 (purple) and 2018 (yellow). Recent studies focused on tissue acquisition, size of the needle, lumen-apposing metal stents, and fine-needle-biopsy.

**TABLE 4:** The top 10 most frequently cited references of EUS research.

Reference	Citations
WIERSEMA MJ (12), GASTROENTEROLOGY, V112, P1087	436
BRUGGE WR (13), GASTROENTEROLOGY, V126, P1330	291
WILLIAMS DB (14), GUT, V44, P720	267
CHANG KJ (20), GASTROINTEST ENDOSC, V45, P387	261
TANAKA M (15), PANCREATOLOGY, V12, P183	250
TANAKA M (16), PANCREATOLOGY, V6, P17	219
CATALANO MF (17), GASTROINTEST ENDOSC, V40, P442	206
HEWITT MJ (18), GASTROINTEST ENDOSC, V75, P319	206
GIOVANNINI M (19), ENDOSCOPY, V27, P171	204
ROSCH T (21), NEW ENGL J MED, V326, P1721	194

## Discussion

In our current study, we retrieved related publications from the WOS Core database and took advantage of bibliometric analysis to explore and identify the trend, hot spots, and the knowledge base associated with EUS. Our findings revealed several intriguing and thought-provoking facts. As already known, the annual number of academic publications is an important indicator of evolving trends in the field. Before 1997, the volume of publications in the EUS field has continuously increased. Beginning with the year 1997, the volume of EUS-related publications has expanded with particularly notable gains in the past decade, which suggested that EUS attracted great research interest. The annual publications reflect increasing interest in this field from researchers. The rapid development of EUS in the past decade is striking. We speculated that the advent of EUS-guided fine-needle-aspiration (EUS-FNA) might contribute to this development in this period (12). Peter Vilmann first performed EUS-FNA for the diagnosis of a pancreatic lesion in 1991. After that, Wiersema et al. and Vilmann et al. separately described upper and lower gastrointestinal tract EUS-FNA in 1992. As a novel technology has developed and the demand for minimally invasive techniques continues to grow, the future trend for continuous increase looks promising.

According to the distribution of countries, we found that United States dominated EUS output in terms of the volume. Although United States produced the highest number of publications related to EUS, Japan and China also contributed a substantial volume to this field. The distribution of institutions coincides with the countries’ data based on the geographical location of the institution. Institutions from United States (MAYO CLIN, INDIANA UNIV, UNIV TEXAS MD ANDERSON CANC CTR) dominated EUS research. BHUTANI MS was the most productive author, and HAWES RH was the most influential author according to the citations of publication. ITOI T, SEO DW, KITANO M, LEVY MJ, YAMAO K, and GIOVANNINI M produced more articles recently, especially SEO DW and KITANO M. Among the journals, GASTROINTESTINAL ENDOSCOPY published the highest number of articles, followed by ENDOSCOPY. High influential references mainly focused on the diagnostic roles of EUS.

Co-cited reference cluster analysis revealed that one publication had the most highly cited references. This publication was related to the assessment of diagnostic accuracy of endosonography-guided fine-needle aspiration biopsy for lymph nodes and gastrointestinal wall lesions. It was reported that the diagnostic accuracy of pancreatic EUS-FNA varied from 84 to 92.9% (22, 23). The sensitivity and specificity of EUS-FNA for the diagnosis of pancreatic neoplasms were 85 and 98% (18), respectively. EUS-FNA had low rates of complications due to its minimally invasive technique and small needle size. Due to the inability to obtain histological architecture, the application of FNA was limited. In order to overcome this limitation, EUS-guided fine needle biopsy (EUS-FNB) was first developed to obtain tissue specimens in the early 2000s, which improved the diagnostic accuracy. For the diagnosis of pancreatic lesions, there were conflicting data among prospective studies for the superiority of EUS-FNA vs. EUS-FNB (4). Both techniques had a relatively high and comparable diagnostic accuracy with a relatively low rate of adverse events. In terms of subepithelial lesions, a recent meta-analysis demonstrated that EUS-FNB outperformed EUS-FNA in the diagnostic performance of gastrointestinal subepithelial lesions (24). For pancreatic cystic lesion, EUS-FNB was proved as a safe and effective tool for acquisition of pancreatic cysts by a meta-analysis (25). Further trials are needed for further elucidation of the clinical benefit of EUS-FNB.

Compared with traditional reviews, our study based on bibliometric tools provided more quantitative and comprehensive information on the research foci, trends, and collaboration, which provided a better insight into the evolving EUS future field. As stated above, there are several limitations that we must acknowledge. Firstly, we only included publications from the WOS database and excluded non-English publications, which may lead to publication bias. Secondly, the citation data might be limited by time since earlier papers had a higher chance of being cited than recent ones. This might have led to the fact that some potentially influential papers could not be cited with high citations due to the short duration.

This study provides a new insight and perspective on the trends of EUS research. It revealed the current trend of global EUS research in the past and current state. A growing interest in EUS is observed globally and an increasing trend of scientific production in this field in the next few years will enlighten this point.

## Data Availability Statement

The raw data supporting the conclusions of this article will be made available by the authors, without undue reservation.

## Author Contributions

XH had the concept for the study. XH, XLC, HH, XC, XQC, ZW, MX, and YF conducted data extraction and statistical analysis. XH, XLC, and HH wrote the first draft of the manuscript. All authors edited and critically revised the final version of the manuscript.

## Conflict of Interest

The authors declare that the research was conducted in the absence of any commercial or financial relationships that could be construed as a potential conflict of interest.

## Publisher’s Note

All claims expressed in this article are solely those of the authors and do not necessarily represent those of their affiliated organizations, or those of the publisher, the editors and the reviewers. Any product that may be evaluated in this article, or claim that may be made by its manufacturer, is not guaranteed or endorsed by the publisher.

## Abbreviations

EUS: Endoscopic ultrasound
WOS: Web of Science
EUS-FNA: Endoscopic ultrasound-guided fine needle aspiration
EUS-FNB: Endoscopic ultrasound-guided fine needle biopsy
