# Supplementary figures and images for: A Bibliometric Analysis of Publications on Endoscopic Ultrasound
Source: Front Med (Lausanne). 2022 Mar 29;9:869004. doi: 10.3389/fmed.2022.869004 (PMC9002052; doi:10.3389/fmed.2022.869004)

Supplemental material

Figure S1. Flow chart of endoscopic ultrasound research inclusion.

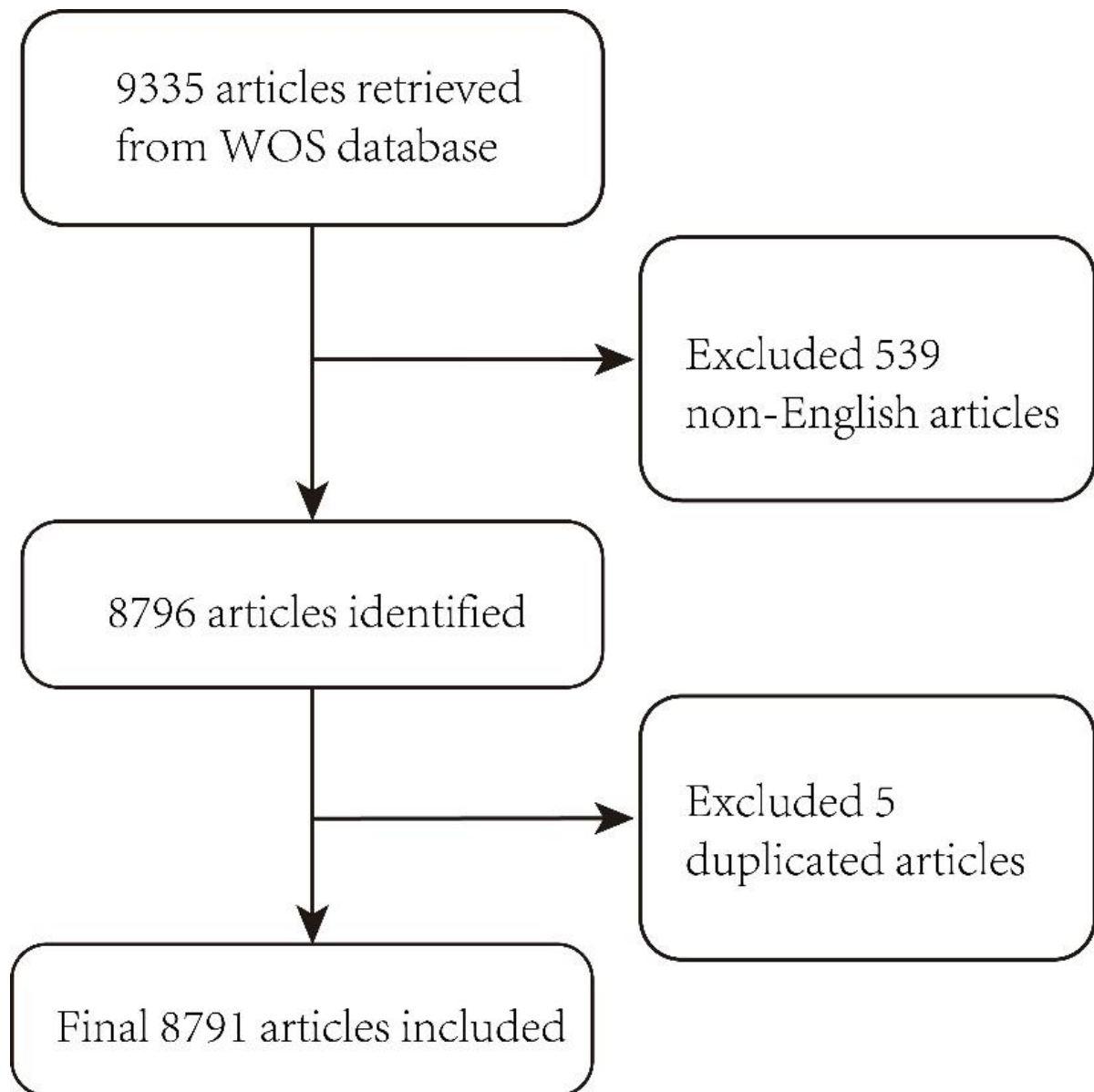

Supplement: Supplementary file 1 [file Image_1.pdf]
